# Loud Auditory Distractors Are More Difficult to Ignore After All: A Preregistered Replication Study With Unexpected Results

**DOI:** 10.1027/1618-3169/a000554

**Published:** 2022-10-18

**Authors:** Lejla Alikadic, Jan Philipp Röer

**Affiliations:** ^1^ Department of Psychology and Psychotherapy, Witten/Herdecke University, Witten, Germany

**Keywords:** irrelevant sound effect, intensity, auditory distraction, attentional capture, working memory

## Abstract

**Abstract.** Working memory performance is markedly disrupted when task-irrelevant sound is played during item presentation or retention. In a preregistered replication study, we systematically examined the role of intensity in two types of auditory distraction. The first type of distraction is the changing-state effect (i.e., increased disruption by changing-state relative to steady-state sequences). The second type is the auditory deviant effect (i.e., increased disruption by auditory deviant relative to steady-state sequences). In previous experiments, the changing-state effect was independent of intensity. Whether a deviation in intensity leads to an increase in disruption has not yet been examined. We replicated the classic finding that the increased disruption by changing-state relative to steady-state sequences is independent of intensity. Contrary to previous studies, we found an unexpected main effect of intensity. Steady-state and changing-state sequences presented at 75 dB(A) were more disruptive than presented at 45 dB(A), suggesting that intensity plays a more important role than previously assumed in the disruption of working memory performance. Furthermore, we tested the prediction of the violation of expectancy account, according to which deviant distractors at a lower and higher intensity than the rest of the sequence should be equally disruptive. Our results were consistent with this prediction.


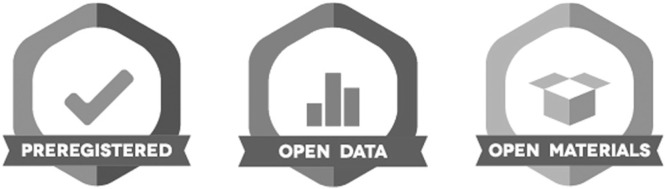


Task-irrelevant, to-be-ignored sound disrupts the retention of information in working memory. Colle and Welsh (1976) were the first to describe the irrelevant sound effect on visual‒verbal serial recall, which has become a standard instrument for investigating auditory distraction in the laboratory (Banbury et al., 2001; Ellermeier & Zimmer, 1997, 2014; Elliott, 2002; Salamé & Baddeley, 1982; Tremblay et al., 2000). In the present study, we will examine two types of auditory distraction: (1) the changing-state effect and (2) the auditory deviant effect. The changing-state effect refers to the increased disruption of serial recall by changing-state sequences relative to steady-state sequences (e.g., Beaman & Jones, 1997; Bell et al., 2017; Jones et al., 1992). A steady-state sequence is a sequence in which the same distractor item is repeated several times, typically a short word or a letter (AAAAAAAAAA). A changing-state sequence is a sequence in which there is a change from one distractor item to the next, for example, natural speech or – more relevant to the present purpose – a sequence of different distractor words (ABCDEFGHIJ). The auditory deviant effect refers to the increased disruption of serial recall by auditory deviant sequences relative to steady-state sequences (e.g., Bell et al., 2019; Hughes et al., 2005, 2007; Vachon et al., 2012, 2017). An auditory deviant sequence is a sequence in which a single distractor item deviates from the rest of the sequence (AAAAAABAAA), for example, because it is presented with a short delay (Hughes et al., 2005) or because it is spoken by a different voice (Hughes et al., 2007).

There is now ample evidence that the disruptive effect of auditory distractors is largely independent of intensity (Colle, 1980; Ellermeier & Hellbrück, 1998; Schlittmeier et al., 2008; Tremblay & Jones, 1999). However, this evidence comes exclusively from studies that examined the role of intensity in the changing-state effect. Whether a sudden increase or decrease in intensity is sufficient to produce an auditory deviant effect has not yet been examined. Thus, it remains an open empirical question to what extent the findings from the changing-state effect can be transferred to the auditory deviant effect. We will address this question in a preregistered replication study examining both effects to provide a more comprehensive understanding of the role of intensity in auditory distraction.

In an exploratory survey (*N* = 39), we asked participants “Imagine you are working on a demanding task that requires your full concentration. Which of the following statements is most likely to be true?” There were three possible answers:

(1) “Low-intensity sounds are more disruptive than high-intensity sounds.”
(2) “Low-intensity and high-intensity sounds are equally disruptive.”
(3) “Low-intensity sounds are less disruptive than high-intensity sounds.”

Thirty-one participants (79%) believed that low-intensity sounds are less disruptive than high-intensity sounds, five participants (13%) that both types of sounds are equally disruptive, and three participants (8%) that low-intensity sounds are more disruptive than high-intensity sounds. These results are very similar to those of Schlittmeier et al. (2008) who compared the disruptive effect of intelligible speech at 55 dB(A) on serial recall with that of intelligible and unintelligible speech at 35 dB(A). After each experimental block, they asked participants to rate how disturbing the auditory distractors were. Although participants subjectively rated the intelligible speech in the high-intensity condition as more disturbing than the intelligible speech in the low-intensity condition, both types of distractor sequences were equally disruptive to serial recall.

Colle (1980) was the first to conduct a study on the effect of intensity using irrelevant speech in the range from 20 to 76 dB(A). Replicating the classical irrelevant speech effect on serial recall, participants made more errors in the conditions with auditory distractors compared to the quiet control condition. Apart from that, there were no differences. The magnitude of disruption was independent of the intensity of the distractors. Ellermeier and Hellbrück (1998) compared the disruptive effect of Japanese speech and instrumental music played at 60 and 75 dB(A) on serial recall performance. In line with Colle (1980), disruption did not increase when the distractors were played at a higher intensity. In four experiments, Tremblay and Jones (1999) investigated the effect of intensity changes within distractor sequences. To this end, steady-state and changing-state sequences were presented in which the distractor items were played either at the same intensity level or at different levels ranging from 55 to 85 dB(A) with the restriction that successive items were not played at the same level, so there was a change in intensity from one item to the next. Changing-state sequences were significantly more disruptive to serial recall compared to steady-state sequences. This was the case for both sequences of spoken consonants (Experiments 1A, 1B, and 2) and sequences of sine-wave tones (Experiment 3). In all four experiments, however, it did not make a difference whether intensity was fixed or changed from one distractor item to the next.

Thus, in summary, it can be said that in none of the seven experiments conducted so far did the intensity of the distractors have any effect on the magnitude of disruption. As mentioned earlier, however, all of these studies only examined the role of intensity in the changing-state effect. Changing-state sequences are characterized by many regular and predictable changes from one distractor item to the next. Auditory deviant sequences, by contrast, are characterized by a single irregular and unpredictable change within the sequence. Especially, sudden loud sounds such as a car horn or a person screaming are known for their attention-grabbing potential. In the laboratory, auditory deviance detection can be measured by the mismatch negativity (MMN) event-related potential (ERP) component (for a review, see Näätänen et al., 2007). Sounds that deviate in intensity from the preceding acoustic context typically elicit such an MMN (Shestopalova et al., 2018), demonstrating that the auditory deviant is detected by the organism. Interestingly, this is not only the case with a sudden increase in intensity (Jacobsen et al., 2003) but also for a sudden decrease in intensity (Althen et al., 2011).

Whether a sudden increase or decrease in intensity disrupts serial recall also has important theoretical implications. According to the duplex-mechanism account of auditory distraction (Hughes, 2014; Hughes et al., 2013), the changing-state effect and the auditory deviant effect represent two fundamentally different forms of distraction. The changing-state effect is due to a conflict between the seriation of the irrelevant sound and the maintenance of the order of the to-be-remembered items, while the auditory deviant effect is the result of a capture mechanism that occurs when a stimulus deviates from the preceding acoustic context. The changing-state effect cannot be controlled but the auditory deviant effect can be. It therefore seems interesting to investigate not only whether a sudden increase or decrease in intensity produces an auditory deviant effect but also whether this effect – should it exist – becomes smaller over the course of the experiment because this would be evidence for habituation, which is considered to be a marker of attentional capture (e.g., Dalton & Hughes, 2014; Vachon et al., 2012).

Previous results highlight the important role of expectancy violations in auditory attentional capture (Nöstl et al., 2012; Röer et al., 2014b; Vachon et al., 2012). Vachon et al. (2012), for example, were able to demonstrate that a voice change after a series of trials in which the distractor sequence was spoken by the same voice produced a marked increase in disruption. At the beginning of an experimental block with a novel voice, however, no such decrease was found, suggesting that it was the violation of a previously built-up expectation that had caused the drop in performance rather than the novelty of the stimulus. In line with this violation of expectancy account, Röer et al. (2014b) found that melodies that unexpectedly changed into a sequence of tone repetitions – giving the impression of a stuck record – were more disruptive than melodies without such repetitions, although the latter contained a larger number of changing states. As in Vachon et al. (2012), this effect gradually became smaller over the course of the experiment, presumably because the tone repetitions became less and less unexpected with each presentation (see also Hughes et al., 2007; Röer et al., 2013). In the present study, we will compare two types of auditory deviant sequences: (1) a sequence in which the deviant distractor is presented at a higher intensity than the rest of the distractors and (2) a sequence in which the deviant distractor is presented at a lower intensity than the rest of the distractors. This will allow us to test the violation of expectancy account provided by Vachon et al. (2012), according to which the disruptive effect of a deviant auditory stimulus is a function of the degree to which it violates previously built-up expectations. From this account, the clear prediction can be derived that both types of auditory deviant sequences should be equally disruptive to serial recall.

The purpose of the proposed study was to conduct a preregistered conceptual replication that investigates the role of intensity in two types of auditory distraction. First, we will attempt to replicate the finding that the changing-state effect is independent of the intensity of the distractors. Second, we will examine whether a sudden increase or decrease in intensity results in an auditory deviant effect. As this study is the first to test this, we will use a straightforward approach and established methodologies. A standard serial recall task and standard distractor sequences will be used which have produced reliable changing-state and auditory deviant effects in previous studies (Röer et al., 2011, 2015). Auditory distractor sequences will be played at 45 dB(A) in the low-intensity condition and at 75 dB(A) in the high-intensity condition, which is approximately equivalent to an eight-fold increase in loudness. We will use a complete within-subject design to minimize error variance associated with individual differences and a large sample size to guarantee sufficient power. If there is an effect of intensity, we will examine whether this effect gradually becomes smaller over the course of the experiment, which would be evidence of habituation.

## Method

### Preregistration Statement

We confirm that the proposed research had not yet been conducted at the time of Stage 1 submission. We have publicly preregistered the accepted protocol on OSF before starting data collection. This protocol is available at the project page on OSF under https://osf.io/6hygj.

### Power Analysis

Of primary interest is the question of whether a sudden change of intensity produces an auditory deviant effect. Thus, the critical test is the comparison between serial recall performance in the steady-state and auditory deviant conditions. A power analysis with G*Power (Faul et al., 2007) showed that given desired error probabilities of α = β = .05, a sample size of *N* = 120 is needed to detect an effect of η_*p*_^2^ = 0.10 (estimated from a recent preregistered replication of the auditory deviant effect using a similar experimental design, Bell et al., 2019).

### Participants

As specified in the preregistration, our aim was to collect as many data sets as possible during the time the laboratory was available. The minimum target sample size was 120 participants. The data from one participant had to be removed because they participated twice. The remaining sample consisted of 130 participants (mean age = 22.9, *SD* of age = 3.9), 87 of whom were female.

### Materials

A standard serial recall task was used. For each trial, eight to-be-remembered digits were sampled randomly without replacement from the set {1, 2, …, 9}. Digits were presented at a rate of 1 Hz (800 ms on, 200 ms off) in black font on a white background in the center of the computer screen.

Auditory distractor words were 12 German one-syllable nouns that are equally frequent in the German language according to the Centre for Lexical Information (CELEX) database (Baayen et al., 1993). With this type of stimulus material, reliable auditory distraction effects have been found in previous experiments (Bell et al., 2012, 2013; Körner et al., 2018). The auditory distractors were recorded digitally at 44.1 kHz using 16-bit encoding. The intensity of the auditory distractors was measured on site in the laboratory using a professional hand-held sound level meter that was inserted through the opening of a polystyrene ear. A loop of the distractors was created and played on the computer that controlled the experiment.

During the presentation of the to-be-remembered digits, six types of auditory distractor sequences (see Table 1 for an overview) were played binaurally over closed headphones with high-insulation hearing protection covers (Beyerdynamic DT 100). In the steady-state condition, the same distractor word was repeated 10 times. In the low-intensity steady-state condition, all distractor words were presented at 45 dB(A), and in the high-intensity steady-state condition, all distractor words were presented at 75 dB(A). In the changing-state condition, 10 different distractor words were presented. In the low-intensity changing-state condition, all distractor words were presented at 45 dB(A), and in the high-intensity changing-state condition, all distractor words were presented at 75 dB(A). In the auditory deviant condition, the same word was repeated 10 times, but the seventh word was presented either with a lower or a higher intensity than the rest of the words. In the low-intensity auditory deviant condition, all distractor words were presented at 45 dB(A), except for the seventh word, which was presented at 75 dB(A). In the high-intensity changing-state condition, all distractor words were presented at 75 dB(A), except for the seventh word, which was presented at 45 dB(A).

**Table 1:** Overview of auditory conditions

Low-intensity steady-state	A A A A A A A A A A
High-intensity steady-state	**A A A A A A A A A A**
Low-intensity changing-state	A B C D E F G H I J
High-intensity changing-state	**A B C D E F G H I J**
Low-intensity auditory deviant	**A A A A A A** A **A A A**
High-intensity auditory deviant	A A A A A A **A** A A A
*Note.* Font weight indicates the intensity at which distractor words were presented, with normal text indicating 45 dB(A) and bold text indicating 75 dB(A).	

### Procedure

Participants were seated in separate cubicles with sound-absorbing walls. Written instructions on the computer screen informed participants that all sound is task-irrelevant and should be ignored. Participants completed a total number of 76 trials, which was divided into two blocks. The training block consisted of four trials without auditory distractors to allow participants to familiarize themselves with the task. The experimental block consisted of 12 trials in each auditory condition (low-intensity steady-state, high-intensity steady-state, low-intensity changing-state, high-intensity changing-state, low-intensity auditory deviant, high-intensity auditory deviant) that was presented in random order. Immediately after the presentation of the to-be-remembered digits, eight question marks appeared on the screen. Participants used the number pad of the keyboard to replace the question marks with the to-be-remembered digits in the order they were presented in. It was possible to skip over a serial position by pressing a *do not know* button on the keyboard, but it was not possible to correct a response. The experiment took 29 min on average.

#### Differences from the Preregistered Method

Due to a technical error, in one of the 76 trials, the auditory distractor sequence was presented at 32-bit instead of 16-bit. We only noticed this after data from 68 participants had already been collected and corrected the error immediately. To test whether this error affected our results, we included a between-subjects factor error (before correction, after correction) in all analyses. The error had no effect on the statistical results, which is why we decided to report the data from all 130 participants in our analyses.

#### Data Availability Statement

All data together with a data dictionary are available at the project page on OSF under https://osf.io/ejx5m/.

#### Confirmatory Analyses

**Figure 1:**
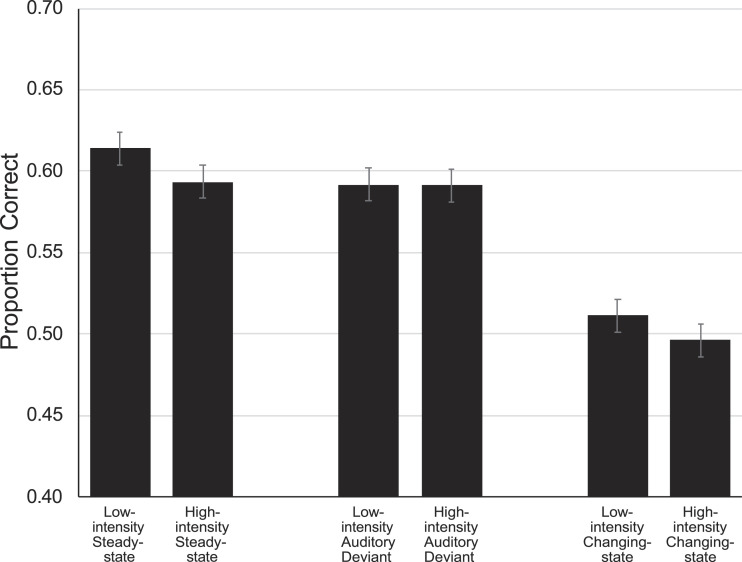
Serial recall performance as a function of auditory distractor condition (low-intensity steady-state, high-intensity steady-state, low-intensity auditory deviant, high-intensity auditory deviant, low-intensity changing-state, high-intensity changing-state). The error bars represent the standard errors of the means.

The key dependent variable was serial recall performance. As specified in the preregistration, responses were scored according to a strict serial-recall criterion (i.e., only digits recalled at the correct serial position will be scored as correct). The most important questions were whether the changing-state effect differs as a function of the intensity of the distractors and whether a sudden increase or decrease in intensity results in an auditory deviant effect. The following analyses were performed to test this. First, we ran a 3 × 2 × 8 repeated-measures MANOVA with auditory condition (steady-state, changing-state, auditory deviant), intensity (low, high), and serial position (1–8) as independent variables and recall performance as dependent variable. We expected to find a significant main effect of auditory condition, and the results were in line with this expectation, *F*(2,128) = 123.34, *p* < .001, η_*p*_^2^ = .66. Unexpectedly, we also found a main effect of intensity, *F*(1,129) = 7.08, *p* = .009, η_*p*_^2^ = .05. There was no interaction of auditory distractor condition and intensity, *F*(2,128) = 1.79, *p* = .172, η_*p*_^2^ = .03. Figure 1 illustrates serial recall performance as a function of auditory distractor condition.

We used orthogonal contrasts on the auditory condition variable and expected performance in the steady-state condition to be better than in the other two conditions combined. In previous experiments, changing-state sequences were more disruptive than auditory deviant sequences (e.g., Bell et al., 2019; Röer et al., 2015). Therefore, we expected to find a similar pattern in our experiment. Both expectations were confirmed by our results. Performance in the steady-state condition was better than in the other two conditions combined, *F* = 131.58, *p* < .001, η_*p*_^2^ = .51, and changing-state sequences were more disruptive than auditory deviant sequences, *F* = 171.73, *p* < .001, η_*p*_^2^ = .57. A first supplementary 2 × 2 × 8 repeated-measures MANOVA with auditory condition (steady-state, changing-state), intensity (low, high), and serial position (1–8) as independent variables and recall performance as dependent variable revealed a changing-state effect evidenced by a main effect of auditory condition, *F*(1,129) = 235.53, *p* < .001, η_*p*_^2^ = .65.

There was also a main effect of intensity, *F*(1,129) = 8.57, *p* = .004, η_*p*_^2^ = .06, but no interaction of auditory distractor condition and intensity, *F*(1,129) = .20, *p* = .654, η_*p*_^2^ = .00. A second supplementary 2 × 2 × 8 repeated-measures MANOVA with auditory condition (steady-state, auditory deviant), intensity (low, high), and serial position (1–8) as independent variables and recall performance as dependent variable revealed an auditory deviant effect evidenced by a main effect of auditory condition, *F*(1,129) = 5.37, *p* = .022, η_*p*_^2^ = .04. There was no effect of intensity, *F*(1,129) = 3.92, *p* = .050, η_*p*_^2^ = .03, and no interaction of auditory distractor condition and intensity, *F*(1,129) = 3.41, *p* = .067, η_*p*_^2^ = . 03.

### Exploratory Analyses

As specified in the preregistration, we also examined whether the changing-state effect and the auditory deviant effect were subject to habituation. To this end, we ran a 2 × 2 × 12 repeated-measures MANOVA with auditory condition (steady-state, changing-state), intensity (low, high), and ordinal trial position (1–12). There was no interaction of auditory distractor condition and ordinal trial position, *F*(11,119) = 1.8, *p* = .059, η_*p*_^2^ = .14; no interaction of intensity and ordinal trial position, *F*(11,119) = 1.28, *p* = .242, η_*p*_^2^ = .11; and no three-way interaction, *F*(11,119) = 1.27, *p* = .252, η_*p*_^2^ = .11. A 2 × 2 × 12 repeated-measures MANOVA with auditory condition (steady-state, auditory deviant), intensity (low, high), and ordinal trial position (1–12) yielded no significant interaction of auditory distractor condition and ordinal trial position, *F*(11,119) = 1.27, *p* = .25, η_*p*_^2^ = .11. The interaction of intensity and ordinal trial position was significant, *F*(11,119) = 2.27, *p* = .015, η_*p*_^2^ = .17, but there was no three-way interaction, *F*(11,119) = .66, *p* = .772, η_*p*_^2^ = .56.

## Discussion

This preregistered replication study yielded an interesting combination of expected and unexpected results. There were three main findings. First, we replicated the finding that the increased disruption by changing-state relative to steady-state sequences (i.e., the changing-state effect) is independent of intensity. Second, there was an overall effect of intensity. Unexpectedly, steady-state and changing-state sequences presented at 75 dB(A) were more disruptive than sequences presented at 45 dB(A). Third, auditory deviant distractors at a lower and higher intensity than the rest of the sequence were equally disruptive. These findings are rather unambiguous and therefore can be interpreted straightforwardly. In all seven experiments conducted so far, the changing-state effect on serial recall was independent of intensity (Colle, 1980; Ellermeier & Hellbrück, 1998; Schlittmeier et al., 2008; Tremblay & Jones, 1999), and this was also the case in the present study. The magnitude of the changing-state effect was comparable, independent of whether the auditory distractors were presented at 45 dB(A) or at 75 dB(A). In the low-intensity condition, serial recall performance in the changing-state condition decreased by 16.7% relative to the steady-state condition, and in the high-intensity condition, performance decreased by 16.4% relative to the steady-state condition. Accordingly, the absolute difference between the steady-state and changing-state condition was the same in both the low- and high-intensity conditions (about 10%) providing further evidence that the magnitude of the changing-state effect is independent of the specific intensity of the distractor sequences.

While we expected to replicate this finding, we did not expect to find an overall effect of intensity. Nevertheless, both steady-state sequences and changing-state sequences presented at 75 dB(A) were clearly more disruptive than the same sequences presented at 45 dB(A). This finding was unexpected because in previous studies, no overall effect of intensity was reported, regardless of the auditory distractor type used (natural speech, music, spoken consonants, sine-wave tones) and of whether the intensity range was high (Colle, 1980; Tremblay & Jones, 1999) or medium to low (Ellermeier & Hellbrück, 1998; Schlittmeier et al., 2008). Therefore, these results seem inconsistent with those from the present experiment at first. On closer inspection, however, the overall pattern of results from previous studies is no longer quite as clear. In Experiments 2 and 3 of Colle (1980), for example, auditory distractors presented at 70 dB(A) were more disruptive at a descriptive level than auditory distractors presented at 40 dB(A) and 50 dB(A), respectively. These data, however, cannot be compared directly, as they come from different experiments, and these experiments “were not designed to evaluate intensity effects” (Colle, 1980, p. 732) and therefore must be interpreted with caution. The interpretation of the evidence in Experiment 1 of Ellermeier and Hellbrück (1998) is not without difficulty, either, because two aspects of the experimental design may have reduced the probability of finding an intensity effect from the outset. First, of all the experiments, the intensity difference between the low-intensity and the high-intensity condition was the smallest in this experiment (i.e., 15 dB[A]), and second, the power to detect an effect was not very large, given a total sample size of only *N* = 12. Still, high-intensity irrelevant speech was more disruptive than low-intensity irrelevant speech descriptively – not only in Experiment 1 of Ellermeier and Hellbrück (1998) but also in Experiment 1 of Schlittmeier et al. (2008) as well where the intensity difference was 20 dB(A) and the total sample size *N* = 20, and thus, the probability of finding an intensity effect was not particularly high from the outset either. In contrast, the results of Tremblay and Jones (1999) are quite unambiguous. In all four experiments, sequences in which the intensity changed from one distractor item to the next were no more disruptive than sequences in which the intensity was fixed (in Experiment 2, they were even less disruptive). On average, however, the intensity in both conditions was the same so that these results do not allow any conclusions to be drawn about an overall intensity effect. In summary, then – although the intensity of auditory distractions has previously been assumed to have a negligible effect on the magnitude of disruption – it may well be that an overall effect of intensity exists, but this effect has remained undetected for the reasons stated above. Furthermore, there was no evidence of a reduction of the intensity effect over the course of the experiment. However, since the overall effect of intensity was an unexpected finding, the experiment was not specifically designed to answer this question. Habituation effects are typically investigated using experimental designs involving a passive listening phase (e.g., Banbury & Berry, 1997; Bell et al., 2012) or designs, in which the same distractor sequence is presented repeatedly (e.g., Beaman & Röer, 2009; Röer et al., 2014a). Although the results seem to suggest that working memory performance does not recover gradually with repeated exposure to loud auditory distractors, it would be interesting to revisit this question in future studies using an experimental design that allows for more sensitive measurements.

The overall intensity effect is also highly relevant from an applied perspective. On the basis of the lack of an intensity effect in previous studies, recommendations have been made such as “minor noise-reduction measures are not likely to be effective” (Ellermeier & Hellbrück, 1998, p. 1409) and “lowering the volume of irrelevant sound is, however, not a viable way forward” (Beaman, 2005, p. 1057). Evidently, these recommendations can no longer be maintained in their absoluteness. While, of course, the acoustic complexity remains the most important factor – our results have confirmed this yet again – intensity must not be overlooked when determining the disruptive potential of a distractor sequence. From a practical point of view, measures to reduce the intensity of background sound can arguably be implemented more rapidly and cost-effectively in many learning and working environments compared to measures to reduce its complexity. Our results show that this in itself can have a positive effect on the retention of information in working memory. After all, the participants in our exploratory survey do not seem to have been so wrong in their assumption that low-intensity sounds are less disruptive than high-intensity sounds. This overall effect of intensity may be investigated more thoroughly in future experiments. It has been shown previously, for example, that intelligible speech presented at 48 dB(A) was significantly more disruptive to serial recall than unintelligible speech of the same intensity (Haapakangas et al., 2008). It cannot be completely ruled out that in the present experiment, the auditory signal at low intensity was masked to some extent by environmental noise. A replication of the experiment in an acoustic anechoic chamber or a systematic manipulation of intelligibility could bring clarity to this possible explanation for the effect.^1^ We also examined whether a deviation in intensity leads to an increase in disruption. Auditory deviant effects have been reported for sequences with unexpected delays in the presentation (Hughes et al., 2005), unexpected voice changes (Hughes et al., 2007), and unexpected distractor repetitions (Marsh et al., 2014).

Although sudden loud sounds are known for their attention-grabbing potential (e.g., auditory alarms, Foley et al., 2020) and an MMN is elicited both following an unpredictable increase in intensity (Jacobsen et al., 2003) and an unpredictable decrease in intensity (Althen et al., 2011), it had not yet been examined whether high-intensity sounds in a sequence of low-intensity sounds and low-intensity sounds in a sequence of high-intensity sounds produce an auditory deviant effect. The violation of expectancy account (Vachon et al., 2012) predicts that both types of sequences should be equally disruptive because the disruptive effect of an auditory deviant is assumed to be a function of the degree to which it violates previously built-up expectations. The results presented here are fully consistent with this assumption.

In summary, it can be said that while we have successfully replicated that the increased disruption by changing-state relative to steady-state sequences is independent of the intensity, this does not mean at the same time that the intensity of auditory distractions is irrelevant. On the contrary, we found clear evidence that high-intensity steady-state and changing-state sequences are more difficult to ignore than their low-intensity counterparts. Thus, the overall intensity should be taken more carefully into account when determining the disruptive potential of auditory distractor sequences in the future, for example, in the design of working and learning environments. Moreover, the intensity deviant effect presented here shows once again that both bottom-up (e.g., overall intensity) and top-down influences (e.g., the degree to which a stimulus violates previously built-up specific expectations about the continuation of the sequence) are important determinants in the disruption of working memory performance, which is consistent with a growing body of recent evidence on auditory distraction (Nöstl et al., 2012; Parmentier et al., 2011; Röer et al., 2019; Vachon et al., 2012, 2020).

## Footnotes

^1^
We thank an anonymous reviewer for making us aware of this possibility.

## Notes

**Open Data:** To the best of my ability and knowledge, we have provided all original materials and clear references to all other materials via a stable online repository.

We confirm that the proposed research had not yet been conducted at the time of Stage 1 submission. We have publicly preregistered the accepted protocol on OSF before starting data collection. This protocol is available at the project page on OSF under https://osf.io/6hygj (Alikadij & Röer, 2021).

The raw data and additional information are deposited at https://osf.io/ejx5m/ (Alikadij & Röer, 2022).
